# Supplementary material for: Implementation of the Obturator Nerve Block into a Supra-Inguinal Fascia Iliaca Compartment Block Based Analgesia Protocol for Hip Arthroscopy: Retrospective Pre-Post Study
Source: Medicina (Kaunas). 2020 Mar 27;56(4):150. doi: 10.3390/medicina56040150 (PMC7230392; doi:10.3390/medicina56040150)
Supplement: Supplementary file 1 [file medicina-56-00150-s001.zip › Figure S1_Binder1.pdf]

Intraoperative Remifentanil dose Data analysis using R

Boohwi Hong

Package install

Data import

Data structure

```
str(d1)
```

```
## 'data.frame':    77 obs. of  6 variables:
##  $ id         : int  1 2 3 4 5 6 7 8 9 10 ...
##  $ group      : Factor w/ 3 levels "F","FO","N": 3 1 1 1 3 3 1 1 3 3 ...
##  $ remi       : int  900 1000 750 1205 1205 1710 1000 1205 1205 1205 ...
##  $ fentanyl_12: num  686 507 345 400 287 ...
##  $ fentanyl_24: num  805 791 636 417 389 ...
##  $ fentanyl_6 : num  219 385 219 341 211 ...
```

Explorative data analysis with graphics

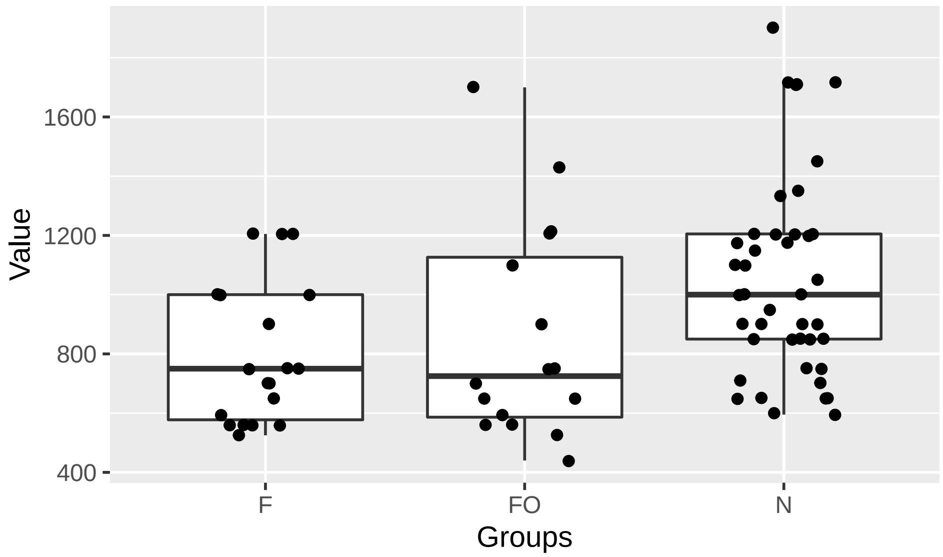

Easystat function developed by S. Park (available at https://rpubs.com/goodlebang)

Statistical Result

```r
easystat(d1)
```

```
## 1. Normality assumption test by Shapiro_Wilk test is
##  p = 0.000
##  Normality assumption was rejected
## 2. The result of Kruskall_Wallis test:
##  p = 0.009
##  A statistically significant difference exist between groups
```

```
## Dunn (1964) Kruskal-Wallis multiple comparison
```

```
##   p-values adjusted with the Benjamini-Hochberg method.
```

```
##   Comparison          Z     P.unadj      P.adj
## 1     F - FO -0.2992455 0.764752738 0.76475274
## 2      F - N -2.7057027 0.006816005 0.02044801
## 3     FO - N -2.2007097 0.027756581 0.04163487
```

Fentanyl dose 6hours Data analysis using R

Boohwi Hong

Package install

Data import

Data structure

```
str(d1)
```

```
## 'data.frame':    77 obs. of  5 variables:
##  $ id         : int  1 2 3 4 5 6 7 8 9 10 ...
##  $ group      : Factor w/ 3 levels "F","FO","N": 3 1 1 1 3 3 1 1 3 3 ...
##  $ fentanyl_6 : num  219 385 219 341 211 ...
##  $ fentanyl_12: num  686 507 345 400 287 ...
##  $ fentanyl_24: num  805 791 636 417 389 ...
```

Explorative data analysis with graphics

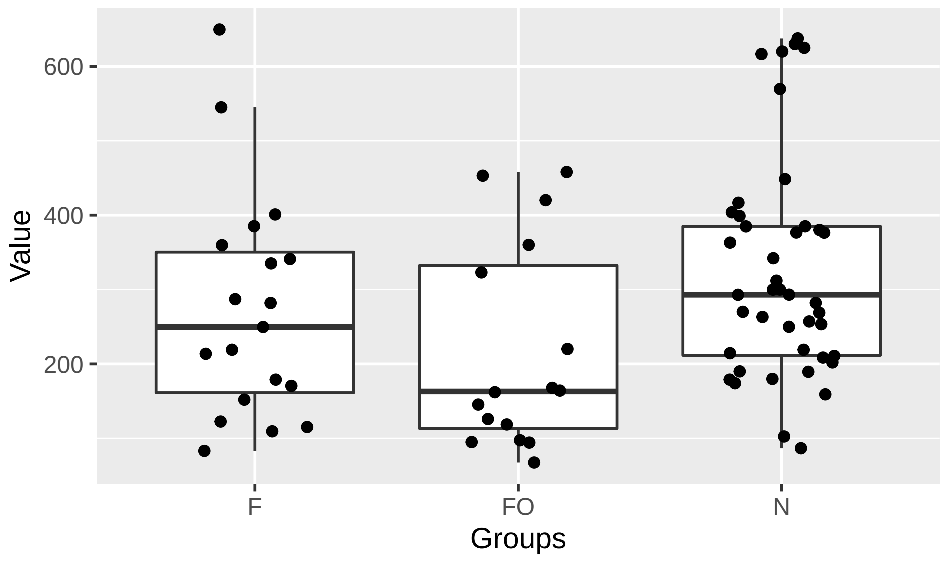

Easystat function developed by S. Park (available at https://rpubs.com/goodlebang)

Statistical Result

```r
easystat(d1)
```

```
## 1. Normality assumption test by Shapiro_Wilk test is
##  p = 0.000
##  Normality assumption was rejected
## 2. The result of Kruskall_Wallis test:
##  p = 0.028
##  A statistically significant difference exist between groups
```

```
## Dunn (1964) Kruskal-Wallis multiple comparison
```

```
##   p-values adjusted with the Benjamini-Hochberg method.
```

```
##   Comparison         Z     P.unadj      P.adj
## 1     F - FO  1.135855 0.256017454 0.25601745
## 2      F - N -1.365690 0.172036161 0.25805424
## 3     FO - N -2.597116 0.009401027 0.02820308
```

Fentanyl dose 12hours Data analysis using R

Boohwi Hong

Package install

Data import

Data structure

```
str(d1)
```

```
## 'data.frame':    77 obs. of  6 variables:
##  $ id         : int  1 2 3 4 5 6 7 8 9 10 ...
##  $ group      : Factor w/ 3 levels "F","FO","N": 3 1 1 1 3 3 1 1 3 3 ...
##  $ fentanyl_12: num  686 507 345 400 287 ...
##  $ X          : logi  NA NA NA NA NA NA ...
##  $ fentanyl_24: num  805 791 636 417 389 ...
##  $ fentanyl_6 : num  219 385 219 341 211 ...
```

Explorative data analysis with graphics

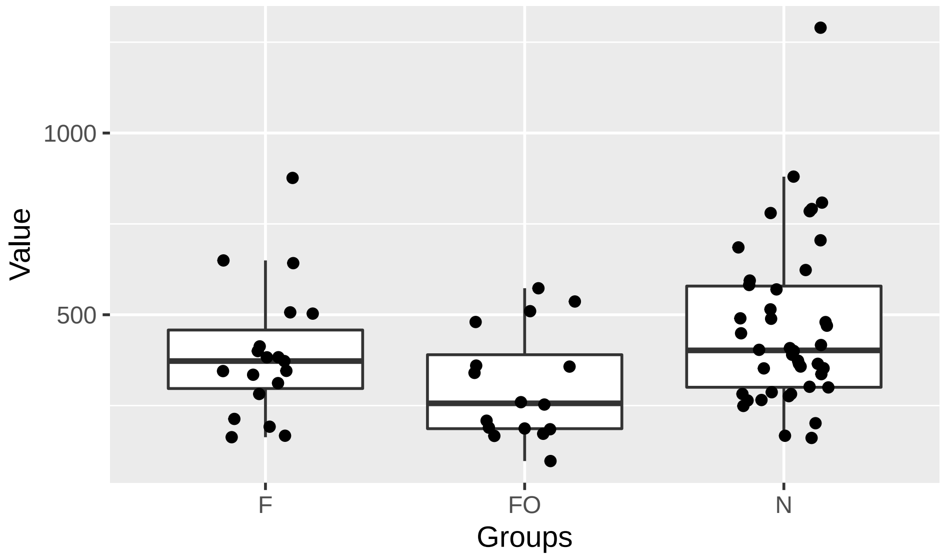

Easystat function developed by S. Park (available at https://rpubs.com/goodlebang)

Statistical Result

```r
easystat(d1)
```

```
## 1. Normality assumption test by Shapiro_Wilk test is
##  p = 0.000
##  Normality assumption was rejected
## 2. The result of Kruskall_Wallis test:
##  p = 0.024
##  A statistically significant difference exist between groups
```

```
## Dunn (1964) Kruskal-Wallis multiple comparison
```

```
##   p-values adjusted with the Benjamini-Hochberg method.
```

```
##   Comparison         Z     P.unadj      P.adj
## 1     F - FO  1.483838 0.137851810 0.20677771
## 2      F - N -1.060351 0.288985096 0.28898510
## 3     FO - N -2.711670 0.006694524 0.02008357
```

Fentanyl dose 24hours Data analysis using R

Boohwi Hong

Package install

Data import

Data structure

```
str(d1)
```

```
## 'data.frame':    77 obs. of  6 variables:
##  $ id         : int  1 2 3 4 5 6 7 8 9 10 ...
##  $ group      : Factor w/ 3 levels "F","FO","N": 3 1 1 1 3 3 1 1 3 3 ...
##  $ fentanyl_24: num  805 791 636 417 389 ...
##  $ fentanyl_12: num  686 507 345 400 287 ...
##  $ X          : logi  NA NA NA NA NA NA ...
##  $ fentanyl_6 : num  219 385 219 341 211 ...
```

Explorative data analysis with graphics

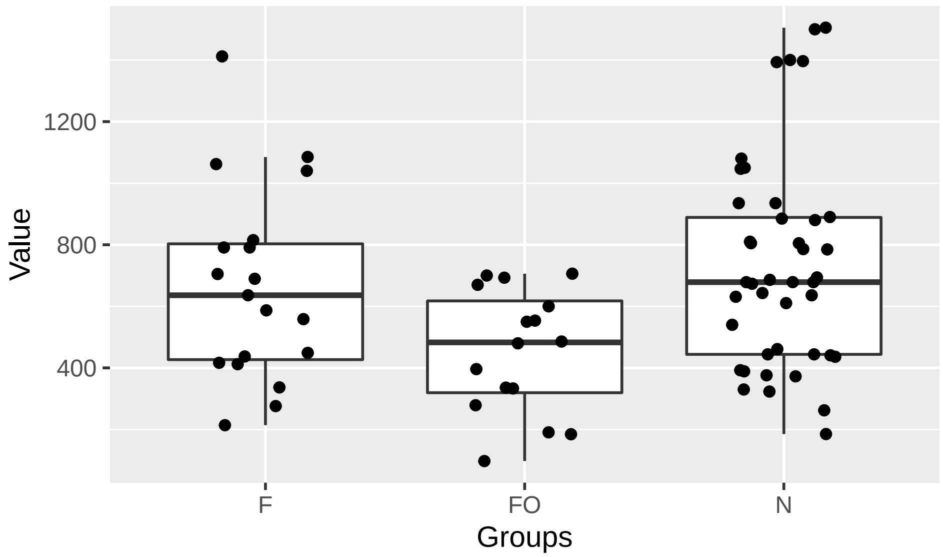

Easystat function developed by S. Park (available at https://rpubs.com/goodlebang)

Statistical Result

```
easystat(d1)
```

```
## 1. Normality assumption test by Shapiro_Wilk test is
##  p = 0.006
##  Normality assumption was rejected
## 2. The result of Kruskall_Wallis test:
##  p = 0.018
##  A statistically significant difference exist between groups
```

```
## Dunn (1964) Kruskal-Wallis multiple comparison
```

```
##   p-values adjusted with the Benjamini-Hochberg method.
```

```
##   Comparison          Z     P.unadj      P.adj
## 1     F - FO  2.0116757 0.044254133 0.06638120
## 2      F - N -0.5270856 0.598134175 0.59813418
## 3     FO - N -2.8194483 0.004810629 0.01443189
```
