# Supplementary figures and images for: Implementation of the Obturator Nerve Block into a Supra-Inguinal Fascia Iliaca Compartment Block Based Analgesia Protocol for Hip Arthroscopy: Retrospective Pre-Post Study
Source: Medicina (Kaunas). 2020 Mar 27;56(4):150. doi: 10.3390/medicina56040150 (PMC7230392; doi:10.3390/medicina56040150)

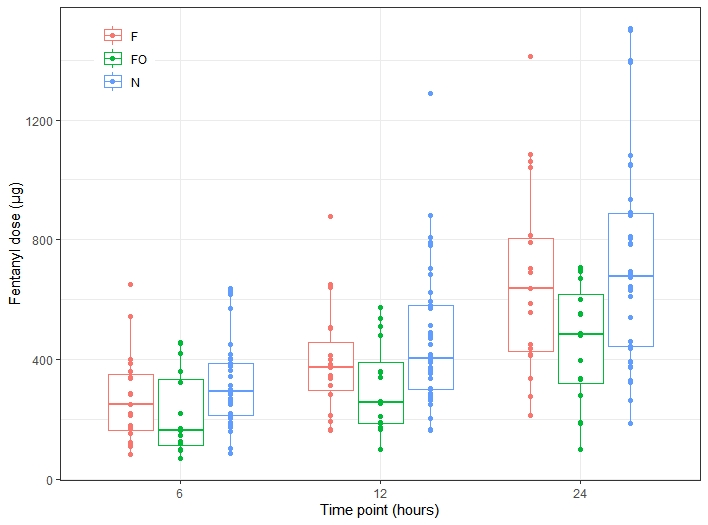

Supplement: Supplementary file 1 [file medicina-56-00150-s001.zip › Figure S2_fentanyl dose plot.jpeg]
